# Anemia and Nutritional Status of Syrian Refugee Mothers and Their Children under Five Years in Greater Beirut, Lebanon

**DOI:** 10.3390/ijerph18136894

**Published:** 2021-06-27

**Authors:** Joana Abou-Rizk, Theresa Jeremias, Lara Nasreddine, Lamis Jomaa, Nahla Hwalla, Hani Tamim, Jan Frank, Veronika Scherbaum

**Affiliations:** 1 Institute of Nutritional Sciences (140), University of Hohenheim, 70599 Stuttgart, Germany; theresa.jeremias@uni-hohenheim.de (T.J.); jan.frank@uni-hohenheim.de (J.F.); veronika.scherbaum@uni-hohenheim.de (V.S.); 2 Department of Nutrition and Food Sciences, American University of Beirut, Beirut 11-0236, Lebanon; ln10@aub.edu.lb (L.N.); lj18@aub.edu.lb (L.J.); nahla@aub.edu.lb (N.H.); 3 Department of Internal Medicine, American University of Beirut, Beirut 11-0236, Lebanon; htamim@aub.edu.lb

**Keywords:** anemia, obesity, maternal nutrition, nutritional inadequacy, pregnancy, lactation, children under five years, Syrian refugees, Lebanon

## Abstract

The objective was to assess the prevalence of anemia and nutritional status of mothers and children under five years among Syrian refugees in Lebanon and to identify nutritional deficiencies among pregnant, lactating, and non-pregnant non-lactating (NPNLM) mothers. A cross-sectional study was conducted among Syrian refugee mothers with children under five years in Greater Beirut, Lebanon (*n* = 433). Data on socio-economic status, maternal health, lifestyle characteristics, dietary intake, anthropometric measurements, and hemoglobin concentrations were collected. The prevalence of anemia was 21.7% among mothers and 30.5% among children. NPNLM with overweight/obesity and an at-risk waist circumference (WC) had 14.7-times and 10.9-times higher odds of anemia than mothers with normal WC and weight. Children of anemic mothers had 2.7-times and 4.4-times higher odds of total and mild anemia than those of non-anemic. Higher odds of mild anemia were found among children of lactating mothers than of NPNLM. A high percent energy intake of total fat and sugar was found among all mothers. Nutritional inadequacy was identified in higher proportions of lactating and pregnant mothers than NPNLM. Our findings highlighted the co-existence of overnutrition and anemia among Syrian refugee mothers and undernutrition among children from the same household. Culture-specific interventions are needed to support maternal nutrition, to ensure the health and wellbeing of their offspring.

## 1. Introduction

The burden of malnutrition remains a worldwide challenge [1]. Globally, nearly two billion adults are estimated to be overweight or obese and more than 140 million children to be stunted [1,2]. According to the World Health Organization (WHO), over half a billion women of reproductive age and 269 million children under five years suffer from anemia [3]. The coexistence of undernutrition (wasting, underweight, stunting, and micronutrient deficiencies), overnutrition (overweight and obesity), and non-communicable diseases within populations, households, and even individuals characterizes the double burden of malnutrition, mostly affecting low- and middle-income countries [4]. Poor economic development and rapid nutrition transition are exposing a growing proportion of the population to unhealthy environmental stressors and health consequences in low- and middle-income countries. Malnutrition in early life has long-lasting effects, such as chronic inflammation, dysbiosis, obesity, non-communicable diseases, stunting, and birth complications [5]. 

Maternal nutritional status at the time of conception and during pregnancy as well as early nutrition during the first two years of life determine to a great extent the health of the offspring, influencing more than a generation through intergenerational cycles [6,7,8]. Optimum maternal and child nutrition during the first 1000 days of life is crucial for brain development and for the prevention of chronic diseases and obesity at later stages of life [7,9,10]. Maternal malnutrition and micronutrient deficiencies may lead to permanent consequences on nutritional programming that can influence children’s growth, development, and survival [6,8,10]. Inadequate quantity and quality of nutrients in the maternal diet can expose women and children to various forms of malnutrition and to increased vulnerability to infections and deficiencies, especially anemia [5,7,10]. Serious health consequences may affect pregnant women and their offspring as a result of anemia, including prematurity, low birth weight, poor health and development, and reduced work productivity [11,12,13]. 

In the Middle East and North Africa region, food security is deteriorating rapidly, primarily driven by conflicts and political instability [14]. Poverty and food insecurity lead to both undernutrition and overnutrition concomitantly [15]. The Eastern Mediterranean Region has been witnessing rapid changes in food consumption habits coupled with a high burden of micronutrient deficiencies, increasing obesity rates, and a persistent burden of undernutrition. Women and girls are particularly prone to various forms of malnutrition in this region [16,17,18]. 

Syria has witnessed the longest and most intense conflict of the *Arab Awakening* resulting in the largest refugee crisis globally, exceeding 6.7 million by the end of 2018. It is estimated that more than 1.5 million Syrian displaced people live in Lebanon with nearly one million who were registered as refugees with the UN High Commission on Refugees (UNHCR) by November 2018, of whom more than half are women and children [19,20]. Lebanon continues to host the highest per capita concentration of refugees worldwide; one out of four people in Lebanon is a displaced person from Syria [21,22]. The conflict in Syria has exacerbated pre-existing development constraints and political instability in Lebanon, in response Syrian refugees have become even more vulnerable. Despite extensive humanitarian assistance, UN reports showed that more than half of Syrian refugee households were still unable to meet survival needs of food, health, and shelter; and one-third remained moderately to severely food insecure in 2018 [23]. 

Even though numerous assessments were conducted by international and national non-governmental organizations (NGOs) on Syrian refugees in Lebanon, there are limited studies measuring the prevalence of anemia and examining the nutritional status of refugees following a humanitarian crisis. Findings were relevant to the undernutrition and anemia status of children and women among Syrian refugees in Lebanon, yet overnutrition and nutritional inadequacies were overlooked [24,25]. Therefore, the objectives of this study were to (1) assess the prevalence of anemia and the nutritional status of Syrian refugee mothers and children under five years, (2) examine the dietary intake of pregnant, lactating, and non-pregnant non-lactating mothers of children below five years of age, and (3) examine associations between anemia, nutritional status, and socio-economic characteristics among Syrian refugee mothers living in Greater Beirut, Lebanon. 

## 2. Materials and Methods

### 2.1. Study Design and Sampling Method

A cross-sectional survey was conducted among mothers and one of their children below five years in the Greater Beirut area in Lebanon. Mother–child dyads were recruited through the Primary Health Care Centers (PHCC), which are part of the National Primary Health Care (PHC) Network overseen by the Ministry of Public Health (MoPH), in 6 vulnerable localities of Greater Beirut, between July and September 2018. A two-step purposeful sampling was used to select recruitment sites located in the most vulnerable areas of Greater Beirut. The catchment area of PHCC located in Greater Beirut encompasses the urban agglomeration of the capital city of Beirut and adjacent districts of Mount Lebanon Governorate. Greater Beirut is the melting pot of the country as its inhabitants consist of more than half of the Lebanese population and over 305,000 refugees [22,26]. The districts were selected according to the highest vulnerability level of localities [27]. The survey areas included in this project are as follows: Baouchriyeh, Bourj Barajneh, Bourj Hammoud, Chiyah, Mazraa, and Mousaytbeh. The identification of PHCC and access to recruitment sites were carried out with the support of the Primary Health Care Department of the MoPH in Lebanon. Selected PHCC from these areas were approached with the approval letter from the MoPH.

**Figure 1:**
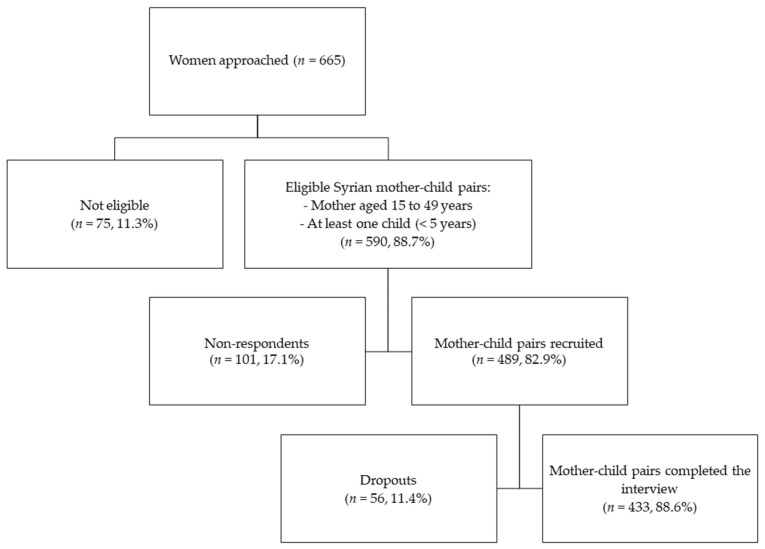
Flowchart of the recruitment subjects in the survey.

Previous estimates of the anemia prevalence among Syrian refugee women (26.1%) and children under five in Lebanon (21.0%) were used for the calculations of the sample size [25]. Based on the highest prevalence found among women, a required sample of 296 mothers was estimated at a 95% confidence interval (95% CI) with a 5% margin of error. The sample size was adjusted with an estimated design effect of 1.5 for the sampling design (N = 444). Considering an estimated non-response rate of 15% and a dropout rate of 10%, a total of 555 mother–child dyads were targeted for the survey. Inclusion criteria for women of reproductive age to participate in this study were: (1) to hold the Syrian nationality, (2) to be between 15 and 49 years old, (3) to have a child aged between 0 and 59 months, (4) for the child to hold the Syrian nationality, and (5) for the child not to suffer from any inborn errors of metabolism or physical malformations. A convenience sample was used to recruit all eligible mothers at the selected centers. When multiple children under five years were eligible within one family, one child was selected randomly. Figure 1 represents the flowchart of the subjects’ recruitment into the survey depicting a non-response rate of 17.1% and a dropout rate of 11.4%. Of the completed interviews, all Syrian pregnant, lactating, and non-pregnant non-lactating mothers with one child below the age of five years were selected for this study (N = 433).

### 2.2. Recruitment Strategy

The recruitment strategy included identifying mothers of children under five years through three approaches: (1) via the assistance of nurses at PHCC, (2) by direct approach from the research assistant in the waiting rooms, or (3) by posting flyers with a short description of the survey in the PHCC premises. The first approach occurred through the nurse or staff who has an established relationship with the mother. The second strategy was deemed necessary given that nurses could be overwhelmed with responsibilities and may not be able to approach and invite mothers to the survey on a regular basis. The third strategy allowed participants to reach out, if interested. Using a developed oral script, nurses and research assistants briefed the potential participant on the content of the survey, checked for the mother and child’s eligibility criteria, and obtained the mother’s informed written consent. 

### 2.3. Data Collection

Face-to-face interviews took place in a private setting at the PHCC premises by Collaborative Institutional Training Initiative (CITI) certified and trained research assistants using a culture-specific multi-component questionnaire. Research assistants underwent an intensive and vigorous one-week training prior to the initiation of field work. This was necessary to obtain a nonjudgmental and neutral attitude, decrease potential social desirability bias, and minimize intra-observer measurement errors. Follow-up training sessions were conducted on a regular basis to maintain the quality of measurements among all enumerators. Data quality control, as well as random cross-checks during data entry, were conducted on questionnaires on a continuous basis, to reduce the risk of reporting bias and to increase the accuracy of the data. Information was collected on characteristics of the household, socio-economic status, maternal lifestyle, and health status. Household monthly income was classified as either being ≤750,000 Lebanese Pounds (LBP) or >750,000 LBP, which was the equivalent of 500 US dollars (USD) at the time of data collection. This classification was based on the legal minimum wage in Lebanon, approximately 675,000 LBP (equivalent to 450 USD in 2018) [27]. The crowding index was used as a proxy measure for the socio-economic status. It was computed as the total number of co-residents per household divided by the number of rooms excluding kitchens, bathrooms, hallways, balconies, and garage according to the American Crowding Index definition [28]. The International Physical Activity Questionnaire Short Form (IPAQ-SF) was used to measure physical activity levels [29]. 

### 2.4. Anthropometric Assessment

Anthropometric measurements were taken by trained research assistants using standardized protocols [30,31] and calibrated equipment. An average of two measurements was recorded to the nearest decimal. Height and weight were measured using a portable mechanical stadiometer (SECA 213) and electronic 2-in-1 weighing scale (SECA 876) with light clothing and bare feet or stockings. Measuring mats (SECA 417) were used to measure the length for children under two years. Mid-upper arm, waist, and hip circumferences were measured for mothers with light clothing using a non-elastic measuring tape (SECA 201). For all mothers, including pregnant mothers, the mid-upper arm circumference (MUAC) score was defined as: <23.0 cm (underweight), 23.0–27.9 cm (normal weight), 28.0–30.9 cm (overweight), and ≥31.0 cm (obese) [32,33,34,35]. Pre-pregnancy weight was not collected from pregnant mothers. For non-pregnant mothers, the nutritional status was evaluated using three indicators: body mass index (BMI), waist circumference (WC), and waist–hip ratio (WHR) as per the WHO classifications. BMI was calculated as the ratio of weight (kg) to height squared (m^2^). Nutritional status was defined as: underweight (BMI < 18.5 kg/m^2^), normal weight (BMI 18.5–24.9 kg/m^2^), overweight (BMI 25.0–29.9 kg/m^2^), and obese (BMI ≥ 30.0 kg/m^2^) [36]. WHR was computed as the ratio of waist circumference by hip circumference. The cut-off points of WC > 80 cm and WHR ≥ 0.85 cm were used to identify “at-risk WC” and “substantially increased risk WHR”, respectively [37]. WC and WHR were used as an indication of increased risk of metabolic complications and abdominal obesity among mothers. For children, the nutritional status was defined using the WHO child growth standards. Length/Height-for-age Z-scores (HAZ) were used to classify children as stunted (HAZ < −2) and not stunted (HAZ ≥ −2). Weight-for-age Z-scores (WAZ) were used to classify children as underweight (WAZ < −2) and not underweight (WAZ ≥ −2). Weight-for-length/height Z-scores (WHZ) were used to classify children as wasted (WHZ < −2) and not wasted (WHZ ≥ −2). BMI-for-age Z-scores (BAZ) were used to classify children as wasted (BAZ < −2), normal weight (−2 ≤ BAZ ≤ 2), and overweight or obese (BAZ > +2) [38]. The WHO Anthro Survey Analyzer was used to derive the *z*-scores [39].

### 2.5. Biochemical Assessment

Members of the research team underwent training on proper micro-technique blood collection for pediatrics and adults in order to measure hemoglobin concentrations (Hb) using the “HemoCue Hb301 System”. Control solutions were routinely used to ensure the accuracy of the measurement. Trained research team members conducted a finger prick on mothers and children (6–59 months) and a heel prick on infants (0–5 months) to collect a small drop of blood. The WHO cut-offs at sea level for determining total anemia were used for lactating and non-pregnant non-lactating mothers as Hb < 12.0 g/dL, and for pregnant mothers and children aged 6 to 59 months as Hb < 11.0 g/dL. Anemia was further classified as mild, moderate, and severe for non-pregnant mothers (11.0–11.9 g/dL, 8.0–10.9 g/dL, and <8.0 g/dL, respectively) and for pregnant mothers and children (6–59 months) (10.0–10.9 g/dL, 7.0–9.9 g/dL, and <7.0 g/dL, respectively) [40]. For infants aged zero to five months, total anemia was defined as Hb < 10.5g/dL, according to Marques et al. (2014) [41] and given the lack of WHO criteria for classifying the severity of anemia for this age group [40]. 

### 2.6. Dietary Assessment

Trained nutritionists within the research team collected data on dietary intake and daily meal patterns. The 2D food portion visuals [42] and standardized reference portions were used to facilitate and standardize the collection of the dietary data. The dietary intake of the mother was measured using the quantitative multiple-pass 24-h dietary recall method. The five-step multiple-pass method starts with the quick uninterrupted listing of foods by the interviewee, proceeds to probing for forgotten foods list and collecting the time and occasion, then a comprehensive description of foods and amounts eaten is gathered in the detailed cycle, and ends with a final probe review [43,44]. Dietary data from the 24-h recalls were analyzed with the NutriSurvey 2007 using the United States Department of Agriculture (USDA) database (SR 28, version: May 2016) [45]. Local single food items were added to the database from local food composition tables [46]. Standardized recipes were used to analyze composite and traditional dishes. 

Dietary intake of the mothers was compared to the Dietary Reference Intakes (DRIs) for energy and nutrients, as recommended by the Institute of Medicine. DRIs refer to the Recommended Dietary Allowances (RDAs), Adequate Intakes (AIs), and Acceptable Macronutrient Distribution Ranges (AMDRs) for energy, macro- and micronutrients [47,48]. Intakes of monounsaturated fatty acids (MUFA), polyunsaturated fatty acids (PUFA), saturated fatty acids (SFA), and trans-fatty acids (TFA) were analyzed based on the FAO recommendations [49]. Cholesterol intake was assessed based on the criteria established by the National Institutes of Health [50]. The reference intake of total sugar was based on the WHO recommendation [51]. Appendix A displays the DRIs for macro- and micronutrients (Table A1) according to their reproductive status. Macronutrients’ intakes were expressed as percent total energy intake (%EI). In addition, the average energy and key macro- and micronutrient intakes were analyzed for mothers, according to their reproductive status. The nutritional inadequacy represents the proportion of mothers not meeting 2/3rd of the RDA or AI for key macro- and micronutrient according to their age group and reproductive status.

### 2.7. Statistical Analysis

Data were entered using KOBO Technology provided by Harvard Humanitarian Institute [52]. Data analysis was carried out using the Statistical Package for Social Sciences, version 27.0 (SPSS Inc., Chicago, IL, USA). Descriptive statistics were performed for continuous and categorical variables. The number of subjects and percentages (*n*, %) represented nominal variables, whereas mean and standard deviation (mean ± SD) were used to represent continuous variables. The reproductive status of the mother was defined as pregnant mothers (PM), non-pregnant lactating mothers (LM), and non-pregnant non-lactating (NPNLM) mothers. Associations were investigated between the reproductive status of the mother and socio-economic and maternal health and lifestyle characteristics, nutritional status of mother and children as well as daily meal patterns of mothers using chi-square analysis for categorical variables and one-way ANOVA test to compare means across groups. Simple and multiple multinomial logistic regressions were used to examine associations of the mother’s reproductive status (dependent variable) with socio-economic and maternal health characteristics, anemia and nutritional status of mothers and children. Logistic regressions were also used to examine the association between maternal anemia (dependent variable) and the nutritional status of mothers and children. Results from the logistic regressions were expressed as OR for crude odds ratios and aOR for adjusted odds ratios with a 95% confidence interval. The significance of each regression model was evaluated using R-squared, the overall percentage, and Hosmer and Lemeshow test. Multicollinearity was measured between all the independent variables within regression models using correlation coefficients, tolerance, variance inflation factors (VIF), and the condition index. Statistical significance was defined as *p*-value < 0.05. Associations close to statistical significance were also reported to improve the interpretation of results [53,54].

## 3. Results

Nearly half of the study sample consisted of LM (47.1%) followed by NPNLM (35.1%) and PM (17.8%). The mean age of mothers was 27.4 years (±5.9) and of children 16.8 months (±14.3). On average, NPNLM were significantly older and LM had the youngest children. The majority of mothers in the study sample were married (98.8%), housewives with no paid job (97.2%) and nearly half of the fathers had no job or a part-time job (48.7%). More than half of the parents had completed primary or intermediate school (mothers: 58.1%; fathers: 68.4%), while 16% were found to be illiterate. Two-thirds of the households (65.5%) had a monthly income below or equal to 750,000 LBP, approximately equal to the legal minimum wage in Lebanon. The majority of the mothers were registered as refugees with the UNHCR (82.1%), of which only 8.9% received food assistance (e-vouchers) from the World Food Programme (WFP). One mother reported receiving food assistance from another source while she was not registered with the UNHCR. Overall, 13.5% of all mothers reported receiving cash or food assistance from the UNHCR or other sources. About half of the respondents lived in nuclear and extended families (52.7% and 47.3%, respectively). A high proportion of households had the father or family-in-law as head of the family (96.2%); in contrast, only 3.8% were headed by the mother or both parents. A mean crowding index score of 3.7 (±1.6) and a mean of three children under five years per household (2.7 ± 1.5) were recorded in our study sample. A significantly higher proportion of PM had one to two children under five years old as compared to LM and NPNLM (Table 1). 

Healthcare service utilization and maternal health characteristics are shown in Table 2. Most mothers were uninsured (93.7%) and about two-thirds of them usually visited public healthcare facilities (69.4%). Overall, 62.1% of the mothers had at least four antenatal care (ANC) visits while 16.6% of the mothers had zero ANC visits during their pregnancy with the child participating in the study. However, PM had the highest proportion of zero ANC visits than LM and NPNLM (21.1%, 16.1%, 15.1%, respectively). A small proportion of the mothers received health and nutrition messages from healthcare professionals exclusively (13.9%) or multiple sources (22.9%), while the majority relied mainly on family, friends, and/or a media platform (63.2%). 

In addition, self-reported previous diagnosis of anemia was highest among PM, who also suffered from higher incidences of flu as compared to other mothers (*p* < 0.05). Thirty percent of the mothers reported using nutritional supplements, of which the majority were PM (62.3%) followed by LM (27.1%) and NPNLM (17.8%). The most consumed supplements were iron and iron-folic acid (65.9%). Vitamin D was significantly more consumed by NPNLM as compared to LM and PM (*p* < 0.001). The reported compliance rate to the use of micronutrient supplements was overall high (80.2%), with the highest being among PM (89.3%).

As for daily meal patterns (Table 3), a significantly lower proportion of NPNLM had a daily breakfast as compared to PM and LM. On the other hand, LM consumed on average a significantly higher number of main meals per day as compared to the other groups of mothers. Coffee and tea consumption were higher during a meal versus between meals among mothers. It was also seen that more LM consumed coffee and tea during a meal compared to the other mothers. A significantly higher proportion of LM had a low-intensity physical activity level, while NPNLM had more high-intensity activity level and higher rates of smoking compared to LM and PM.

The prevalence of anemia among mothers was 21.7%, with 15.4% being mild and 6.3% moderate cases (Table 4). Children aged zero to 59 months showed a higher prevalence of anemia (30.5%), with 24.2% being mild and 9.8% moderate cases. Severe anemia was not detected among both mothers and children. The highest prevalence of total anemia among mothers was observed with NPNLM (26.5%), followed by LM (19.4%) and PM (18.2%). Mild anemia was found the highest among NPNLM (18.5%) and moderate anemia among PM (10.4%). In contrast, the highest prevalence of anemia among children aged zero to 59 months was observed in households of LM (35.6%), followed by NPNLM (27.8%) and PM (22.1%). In addition, mild and moderate anemia among children aged 6 to 59 months were highest among children of LM (37.5% and 12.5%, respectively) as compared to children of PM and NPNLM. A significant association between the reproductive status and the classification of anemia among mothers and children was found, but not with total anemia. As for the nutritional status, overweight and obesity rates were found to be high according to the BMI of non-pregnant mothers (31.2% and 29.5%, respectively) and the MUAC classification among all mothers (26.9% and 34.6%, respectively). In addition, nearly two out of three non-pregnant mothers were found to have an at-risk waist circumference (65.2%). Yet, 9.0% of their children were found to be stunted (HAZ < −2), 4.4% underweight (WAZ < −2), 5.1% wasted (WHZ < −2), and 4.4% overweight or obese (BAZ > +2).

Further analysis showed that moderate- and high-intensity activity levels of mothers were significantly associated with the age of the mother and child and having more children under the age of five years (*p* < 0.05). No association was found between overweight and obesity and their physical activity levels (data not shown).

Multinomial logistic regression analysis (Table 5) showed that PM were more likely to be younger compared to NPNLM, while LM were more likely to have younger children. Higher odds of self-reported anemia and suffering from the flu were found among PM as compared to NPNLM. PM and LM had 12-times and 2-times higher odds of using micronutrient supplements than NPNLM (*p* < 0.05). PM and LM also had higher odds of consuming a daily breakfast and lower odds of smoking were found when compared to NPNLM. Regression analysis showed that the risk of mild anemia was significantly lower among PM (aOR = 0.26, 95% CI: 0.08–0.82); in contrast, the risk of moderate anemia was greater among PM even though it did not reach statistical significance. As for children, the odds of mild anemia were three-times higher among children of LM in comparison to those of NPNLM (*p* < 0.05), while the odds of being wasted (BAZ z-score < −2) were higher for children of PM as compared to those of NPNLM (aOR = 5.90, 95% CI: 1.02–34.18). The odds of total anemia among children (zero to 59 months) were two-times higher among children of LM than those of NPNLM, only after adjusting for confounders. 

Table 6 displays the descriptive characteristics of Syrian refugee mothers by anemia status and their associations with maternal anemia (anemic vs. not anemic) using simple and multiple logistic regressions. Significant associations were found between anemia and the crowding index, WFP food assistance (e-vouchers), and the total number of children using the simple logistic regressions. However, these associations lost their statistical significance after adjusting for confounders. As for the health characteristics, higher odds of self-reported previous diagnosis of anemia were found among anemic mothers as compared to non-anemic mothers (aOR = 2.27, 95% CI: 1.14–4.52). Receiving health and nutrition messages from family, friends, and/or media or from multiple sources was significantly associated with lower odds of anemia. Simple and multiple logistic regressions showed that the odds of total anemia among children aged zero to 59 months increased by nearly 3-folds (aOR = 2.67, 95% CI: 1.42–5.02) among anemic mothers as compared to non-anemic. Mild anemia among children was associated with anemia among mothers only after adjusting for confounders. As for the nutritional status of mothers, non-pregnant mothers with an at-risk waist circumference were 3-times more likely to be anemic (aOR = 3.05, 95% CI: 1.34–6.92) as compared to not anemic non-pregnant mothers. Remarkably, the odds of an at-risk WC and of being overweight or obese were 11-times and 15-times higher among anemic NPNLM compared to non-anemic NPNLM, respectively (*p* < 0.01). The odds of being underweight were 13-times higher among anemic NPNLM as compared to those with normal weight, only after adjusting for socio-economic characteristics. The additional analysis did not find any associations between the consumption of coffee and tea and anemia status among mothers.

Table 7 presents the dietary intake and nutritional inadequacy (<2/3rd of DRIs) of macro- and micronutrients of PM, LM, and NPNLM among Syrian refugees from vulnerable areas of Greater Beirut. The percent energy intake (%EI) of carbohydrates, protein, linoleic and linolenic acid, saturated and trans-fatty acids, and dietary intake of cholesterol were within the AMDRs, even though, on average, all mothers did not meet their energy intake requirement. On the other hand, the %EI of total fat, polyunsaturated fatty acids, and total sugar exceeded on average the recommendations for women. Significant differences in dietary intake and nutritional inadequacy were observed among mothers according to their reproductive status. Despite a significantly higher intake of carbohydrate (g/d) among PM and LM, a higher proportion of PM and LM (20.8% and 29.4%) did not meet 2/3rd of the RDA for carbohydrate intake as compared to NPNLM (13.8%). Similarly, a significantly larger proportion of PM and LM fell below 2/3rd of the RDA for protein than NPNLM (59.7%, 59.3%, and 38.2%, respectively; *p* < 0.05). On the other hand, LM had a higher proportion of nutritional inadequacy for dietary fibers as compared to PM and NPNLM (72.5%, 66.2%, and 70.9%, respectively; *p* < 0.05).

With regards to micronutrients, at least three out of four mothers (75.0% to 93.5%) did not meet 2/3rd of the RDA or AI for potassium, vitamin A, calcium, vitamin E, folate, pantothenic acid, vitamin B12, and vitamin B6. Furthermore, at least half of mothers had intakes of zinc (71.8%), magnesium (69.2%), iron (63.9%), vitamin C (63.0%), and riboflavin (50.5%) below 2/3rd of the RDAs. Interestingly, all mothers did not consume at least 2/3rd of the RDA for vitamin D with an average intake of 0.64 µg/d. A significantly higher proportion of LM did not meet 2/3rd of the RDAs or AIs for potassium, vitamin A, vitamin E, vitamin B6, pantothenic acid, zinc, copper, and manganese, compared to PM and NPNLM. On the other hand, a higher proportion of PM, followed by NPNLM, did not reach 2/3rd of the RDA for iron as compared to LM (81.8%, 80.1%. and 45.1%, respectively; *p* < 0.05) despite the highest intake of total iron being observed among PM (PW: 10.2 ± 9.8; LW: 8.2 ± 6.2; NPNLM: 8.5 ± 7.0 mg/d). 

## 4. Discussion

To our knowledge, this is the first study to examine the prevalence and key determinants of anemia and the nutritional status of Syrian refugee mothers and their children under five years according to the mother’s reproductive status. Overall, the prevalence of anemia was 21.7% among mothers and 30.5% among children under five years, this being categorized as a moderate public health significance according to the WHO classification [55]. Our findings remained below the global prevalence of anemia estimated at 29.9% for women of reproductive age and 39.8% for children under five years in 2019. Nevertheless, an increasing trend of anemia has been recorded by the WHO between 2012 and 2019 among women of reproductive age and children under five years in Lebanon and Syria [3]. Anemia levels reported in the present study were higher than those in previous reports among Syrian refugees in Lebanon, but lower than Iraqi refugees in Lebanon and Syrian refugees in the Za’atari refugee camp in Jordan [24,25,56]. According to a survey conducted in Syria in 2015 and 2016, almost a quarter of women of reproductive age (24.5%) and children under five years (25.9%) had anemia [57]. Our findings are more consistent with anemia levels of the Lebanese host population for women of reproductive age and children under five years about two decades ago [58,59,60,61].

The highest prevalence of total and mild anemia among mothers was noted among non-pregnant non-lactating mothers (NPNLM), followed by lactating mothers (LM) and last by pregnant mothers (PM). Concomitantly, NPNLM were also found to be significantly older than LM and PM. An interpretation of these findings suggests that the occurrence of anemia may increase as women are getting older and move forward in the stages of the life cycle [62], even though maternal age and the number of children under five years were not significantly associated with maternal anemia in our survey. Similar findings were reported among pregnant women in rural Jordan [63]. In contrast, other studies in the literature indicated that the anemia prevalence among pregnant women increased with age and the number of children under five years in the household [62,64]. This is due to increased nutrient requirements during pregnancy and lactation and gradual depletion of the iron stores with repeated pregnancies, although lactation amenorrhea compensates for the loss of iron [10,65,66]. Low maternal iron stores at conception are a strong predictor of the risk of iron deficiency and anemia during pregnancy, but also mirror the infant’s iron endowment after birth [67,68]. In fact, our findings showed that child anemia was strongly associated with maternal anemia. Particularly, children of LM suffered the most from total, mild, and moderate anemia and had 3-times higher odds of mild anemia than those of NPNLM. Possible explanations for the increased risk of child anemia include: (1) an inadequate iron intake of women during pregnancy resulting in a minimized accumulation of iron in the fetus during the last trimester [67,68] and (2) a suboptimal maternal and child nutrition due to increased nutrient requirements leading to greater risks of malnutrition and micronutrient deficiencies [9,10]. Birth spacing and family planning play an important role in replenishing depleted maternal iron stores following a pregnancy [66,69].

The prevalence of anemia among PM in our survey was much lower than the WHO Global and Eastern Mediterranean Region estimates as well as other reported anemia rates from neighboring countries [3,62,63]. Indeed, a significantly lower risk of mild anemia was found among PM in our study (aOR = 0.26, 95%CI: 0.08–0.82). This may be explained by the fact that PM and LM had 12-folds and 2-folds higher odds of using micronutrient supplements than NPNLM in our study, despite an overall low usage of micronutrient supplements by mothers in the study sample. The benefits of multiple ANC visits and micronutrient supplementation, including iron and iron-folic acid, on anemia levels during pregnancy and birth outcomes have been well documented [64,65,68,69]. The access to free or subsidized healthcare assistance in place for UNHCR-registered and unregistered refugees in Lebanon could have played a role in reducing the mild anemia prevalence among PM. As a matter of fact, the majority of Syrian refugees across the nation and in our survey reported having access to primary healthcare services, despite main barriers such as economic vulnerability, area of residence, and household composition [70]. Albeit the high proportion of mothers who attended at least four ANC visits (62.1%), slightly less than a quarter of PM had zero visits in our survey. One explanation can be related to their UNHCR registration status as refugees. It was observed that nearly a quarter of PM were not registered as refugees and had a shorter length of stay in Lebanon as compared to LM and NPNLM, suggesting these women arrived in Lebanon after the suspension of UNHCR registrations by the government in 2015 [70,71]. As a consequence, unregistered PM, with higher nutritional needs and requirements, may not have had access to any cash or food assistance and may have faced additional barriers to access and utilize the free or subsidized healthcare assistance in place for registered and unregistered refugees in Lebanon, such as the lack of awareness of service availability [72]. Multiple sources of health messages, including family, friends, and social media, had a protective impact on maternal anemia. These findings shed light on the crucial role of counselling approaches and awareness-raising sensitive to cultural norms which need to target mothers and older members of the household to improve the health and nutritional status of women of reproductive age. For instance, women’s groups at a community level can be developed to promote the engagement of women in discussions and provide maternal social support [73,74,75].

Medium levels of wasting and low levels of stunting were identified among children under five years as per the WHO classification for malnutrition [76]. These levels were also within observed ranges in the Eastern Mediterranean Region. In contrast, the prevalence of overweight and obesity among children in our study was far below those of countries of the region [77]. Stunting rates have dropped by nearly 40% in the Eastern Mediterranean Region from 1990, despite the milder reduction rate observed since 2012 [78]. This trend is also reflected among the Syrian refugee pediatric population in Lebanon. When comparing our findings to the nutritional assessment conducted by UNICEF in 2013, stunting levels dropped by half, yet wasting and underweight increased sharply [25]. A significantly higher risk of wasting was also found among children of PM in our survey. As stunting indicates chronic malnutrition [79], it suggests that poor dietary habits and food-related coping strategies have been in place for a longer period of time [80].

On the other hand, overnutrition was predominant among non-pregnant mothers with more than 60% being overweight or obese. Our findings are in accordance with the 2016 age-standardized estimates of overweight and obesity in Lebanon and Syria [81] and with previous studies conducted among Lebanese women [82,83]. Even though two-thirds of the mothers appeared to have moderate or high activity levels, no association was found with the nutritional status of mothers in the context of our study. According to Lee et al., the IPAQ-SF typically overestimated physical activity levels by 84% [84]. Moderate to high activity was shown to have a protective role against the risk of obesity among Lebanese women, however, this was in the setting of a better socio-economic status and higher education enabling them to buy food with available resources and the knowledge to make better food and lifestyle choices [85]. With two-thirds of the participants reporting a monthly household income below the legal minimum wage in Lebanon, we project that excess overweight and obesity may be due not only to the shift in dietary patterns, but also to increased food-coping strategies due to food insecurity, thus leading to inadequate diet quality that may be energy-dense yet poor in micronutrients [5,86]. Lebanon has been undergoing the nutrition transition with increasing rates of obesity and non-communicable diseases with a shift in dietary patterns characterized by an elevated intake of foods with high energy density and rich in fat and added sugar [87,88]. The prices of food influence to a great extent the choices of food, and lower socio-economic groups tend to resort to cheaper lower-quality diets [89]. In addition, the interrelationship between economic vulnerabilities, food insecurity, and psychological distress has been well documented and can negatively impact health status [28,90].

Findings from this study demonstrated a significant association between total anemia and overweight or obesity (BMI ≥ 25.0 kg/m^2^) and an at-risk waist circumference among mothers, suggesting the existence of a double burden of malnutrition at the individual level [91]. Controversial findings regarding the relationship of anemia with overweight and obesity, central obesity, and obesity-associated inflammation were found in the literature [92,93]. For instance, Wirth et al. found associations between obesity and inflammation, but showed that the risk of anemia was lower among overweight and obese women [92]. However, obesity is generally linked to micronutrient deficiencies interceded by poor nutrition and chronic inflammation [5,66,94]. Another explanation comes into play linking poverty, food insecurity, and malnutrition including anemia [95] and obesity [82,96]. These findings point towards a poor diet leading to the gradual accumulation of malnutrition, nutrient deficiencies, and anemia in the long term [94]. Maternal obesity, associated with poor micronutrient status, may also impair offspring development and early growth as a consequence of the intergenerational effects of malnutrition [5].

Looking at the dietary intake of mothers, overall low energy intakes were reported by our study population. The latter may be explained by the commonly identified underreporting among women [97]. Misreporting may be biased towards reporting lower intakes of fat, carbohydrates, and alcohol [98] and may be influenced by several psychosocial factors such as being overweight, body image concerns, demographics, depression, anxiety, and social desirability [99]. Nevertheless, the diet of women of reproductive age in our study was characterized by a percent energy intake of total fat and total sugar exceeding their respective recommendations, in addition to inadequate intakes of protein and fibers. Protein-rich foods include meat, poultry, seafood, eggs, milk, and dairy products from animal sources and beans, peas, nuts, and seeds from plant sources. These sources also contribute to the dietary intake of B vitamins, vitamins D and E, iron, zinc, calcium, and magnesium among others. In addition, dietary fibers naturally occur in fruits, vegetables, beans, and nuts as well as whole-grain products. These foods are also known to be the primary contributors to a wide range of vitamins and minerals, namely vitamins A and C, folate, and potassium [100]. As a result, it is not surprising that a widespread inadequate dietary intake of the abovementioned vitamins and minerals was simultaneously observed in the diet of the mothers in our survey. Our findings corroborate with those of Nasreddine et al. showing that Lebanese women had a high intake of total fat and higher consumption of sweets, while a lower micronutrient intake was observed [18]. We suggest that similar dietary patterns, that mimic those of the local population, were a sign of “dietary acculturation”. The latter refers to the adoption and integration of new eating patterns and food choices of the host country after migration [101]. Studies among asylum seekers in Germany, including Syrian refugees, established similar conclusions [102]. Multidimensional factors led to dietary adaptations and a remarkable shift towards Western/urban eating habits among asylum seekers in Germany [103].

Poor nutritional adequacies were also found among mothers according to their reproductive status. Of particular interest are the essential hemopoietic nutrients recognized for their role in erythropoiesis, including protein, iron, vitamins A, B12, B6, C, D, E, folate, riboflavin, copper, and zinc [65,94,104]. Higher proportions of PM and LM did not meet 2/3rd RDA for protein than NPNLM. This is mainly due to their higher requirements, even though no significant differences were found in their daily intake of protein. Concomitantly, the majority of PM and NPNLM fell short of 2/3rd RDA for iron as compared to LM. Since no significant differences were observed in the dietary intake of iron among mothers, the advantage was rather the result of lower needs of iron for LM justified by the lactation amenorrhea [66]. Additionally, LM were the least to reach 2/3rd of the RDA for dietary fibers due to higher requirements compared to PM and NPNLM, even though LM consumed a significantly higher number of meals on average per day. In fact, significantly higher proportions of nutritional inadequacies were found among LM, followed by NPNLM and PM, particularly for vitamin A, vitamin B6, zinc, copper, potassium, and manganese. A suboptimal maternal diet during lactation compromises the human milk quality as the content of certain micronutrients are affected, namely the water-soluble vitamins and fat content [105,106]. Breastfeeding has a vital role in the prevention of all forms of malnutrition and micronutrient deficiencies, indicating the need to improve maternal nutrition and women’s nutritional status as early as possible [107]. Similar to our findings, studies in Lebanon found that women were at risk of micronutrient inadequacies especially for hematinic nutrients including iron, folate, zinc, vitamin B12, and calcium [18,58]. Our findings are also in line with the literature showing low intakes of micronutrients among pregnant women and women of reproductive age in low- and middle-income countries [108,109] and in developed countries [110].

Healthier lifestyle patterns were present significantly more among PM and LM with higher odds of consuming daily breakfast and lower odds of smoking. Our findings are less pronounced compared to a previous assessment conducted among Lebanese pregnant women. These women were more likely to have low birth weight babies and reported a lower duration of breastfeeding [111]. The consumption of coffee and tea during a meal was found to be common among mothers in our survey, especially among LM. However, no association with anemia was found in our study despite that they are potent inhibitors of iron absorption. It can be explained by the multifactorial nature of the causes of and contributors to anemia, such as poor dietary practices, infections and inflammation, and socio-economic factors [65]. In addition, no associations between anemia and socio-economic characteristics were found in our study. This is in accordance with previous studies among women in Lebanon and rural Jordan [58,61,63] and the NHANES study among pregnant women [112].

Shifts towards unhealthy foods and lifestyle, with an escalating burden of overweight and obesity, coexisting with undernutrition and anemia, reflect the nutrition situation of most low- and middle-income countries and countries in the Eastern Mediterranean Region [16]. Despite the extensive humanitarian assistance provided to Syrian refugees in Lebanon since the beginning of the conflict in 2011 [23], Beirut and Mount Lebanon governorates have the fewest number of beneficiaries as compared to other regions in Lebanon [70]. The aforementioned was also reflected in our survey as 82.1% of the Syrian households in Greater Beirut were registered as refugees with the UNHCR, of which only 8.9% were receiving food assistance from the World Food Programme. The nutritional status of vulnerable population groups can be further exposed to acute and chronic malnutrition due to the severe financial crisis in Lebanon since October 2019, resulting in hyperinflation of food prices, soaring unemployment rates, and reduced incomes, combined with containment measures for COVID-19 pandemic and the aftermath of the Beirut port explosion in August 2020 [78,113,114].

The findings of this study should be considered in light of a few limitations. The cross-sectional design allows for the investigation of associations rather than causal relationships. Furthermore, dietary data were explored using one 24-h dietary recall which may face potential limitations due to reliance on memory and day-to-day variations; however, every attempt was exerted to minimize misreporting. The five-step multiple pass recall method may reduce bias and provide accurate estimates of food intake at the population level; however, underreporting under field conditions and its accuracy in overweight and obese persons remain underexplored [43,115]. Nevertheless, the 24-h dietary recall presents several strengths such as capturing all eating patterns and preparation methods, not relying on the literacy of the respondent, not affecting food choices, and having a low burden on the memory [116]. The IPAQ-SF was shown to overestimate levels of physical activity by 84% on average and its use as an indicator of relative or absolute physical activity is weak [84]. Lastly, our study sample consists of Syrian mothers and children under five years attending primary healthcare centers in the most vulnerable areas of Greater Beirut and thus the results cannot be generalized to all Syrian refugees in Lebanon. In the context of this study, a randomized recruitment strategy through the UNCHR could have been more representative of Greater Beirut, yet a convenience sample was selected in order to avoid coercion due to offered benefits to registered refugees and fear related to their legal residency status. One of the strengths of this study is the inclusion of unregistered Syrian refugees and displaced persons in the study population. The number of unregistered Syrian refugees may have increased in Lebanon due to the governmental decision in 2015 to suspend registration of Syrian refugees by the UHNCR or to other associated barriers to obtain legal residency [70,71]. Recruitment took place in primary healthcare centers where patients come freely, without expectations, to participate in vaccination campaigns and/or benefit from other health services. Therefore, our sample population is representative of Syrian refugee mothers attending primary healthcare centers in Greater Beirut. Our population group, mothers with children under five years in a highly vulnerable setting, represents a group of interest for international humanitarian assistance and policy makers at national and regional levels, especially as the number of refugees and displaced people might increase rapidly with current economic crises.

## 5. Conclusions

This study highlights the co-existence of overweight, obesity, and anemia among women of reproductive age as well as undernutrition among children under five years within the same households. Evidence on poor dietary intake and nutritional inadequacies of Syrian refugee mothers during pregnancy and lactation is also presented. Findings from this study can serve as a basis for culture-specific interventions, long-term strategies, and policies to protect and promote maternal and child nutrition for refugees in Lebanon and neighboring countries. The burden of malnutrition and nutritional inadequacies calls for immediate double-duty actions and reforms in order to alleviate poverty and food insecurity and promote more decent and sustainable livelihoods for Syrian refugees [117]. Culture-specific interventions to improve the nutritional status of women of reproductive age and children are crucial, in addition to efforts to protect and promote breastfeeding [16,17]. Family planning services should be intensified to allow the replenishment of nutrient stores by the promotion of birth spacing, reduction of unplanned pregnancies, and receiving adequate nutrition during pregnancy that is essential for the mothers and their offspring’s health and wellbeing [69,109]. Further research is still needed to explore the determinants of the double burden of malnutrition and anemia among refugee women and children in countries affected by protracted conflicts and displacement.

## Figures and Tables

**Table 1:** Socio-economic and household characteristics by the reproductive status of Syrian refugee mothers.

Variables ^a^	PM *n* = 77	LM *n* = 204	NPNLM *n* = 152	Total ^b^ *n* = 433	*p*-Value ^c^
**Socio-economic characteristics**					
**Age of the mothers (years)**	27.0 ± 5.2	26.7 ± 5.8	28.7 ± 6.1	27.4 ± 5.9	**0.005** **0.013**
<25 years	32 (41.6)	90 (44.1)	48 (31.8)	170 (39.4)	
25 to 29 years	18 (23.4)	59 (28.9)	41 (27.2)	118 (27.3)	
30 to 34 years	22 (28.6)	39 (19.1)	35 (23.2)	96 (22.2)	
≥35 years	5 (6.5)	16 (7.8)	27 (17.9)	48 (11.1)	
**Age of the children (months)**	23.0 ± 13.0	7.6 ± 6.3	25.9 ± 15.1	16.7 ± 14.3	**<0.001** **<0.001**
<6 months	4 (5.2)	100 (49.0)	10 (6.6)	114 (26.3)	
6 to 23 months	41 (53.2)	99 (48.5)	75 (49.3)	215 (49.7)	
24 to 59 months	32 (41.6)	5 (2.5)	67 (44.1)	104 (24.0)	
**Child’s sex**					0.372
Male	35 (46.1)	107 (52.5)	85 (55.9)	227 (52.5)	
Female	41 (53.9)	97 (47.5)	67 (44.1)	205 (47.5)	
**Marital status of the mother**					0.948
Engaged/ Divorced/Widowed	1 (1.3)	2 (1.0)	2 (1.3)	5 (1.2)	
Married	75 (98.7)	202 (99.0)	150 (98.7)	427 (98.8)	
**Mother’s education level**					0.519
No schooling/Illiterate	9 (11.7)	35 (17.2)	26 (17.4)	70 (16.3)	
Primary, Intermediate school	44 (57.1)	122 (60.1)	83 (55.7)	249 (58.1)	
Secondary school and higher	24 (31.2)	46 (22.7)	40 (26.8)	110 (25.6)	
**Father’s education level**					0.401
No schooling/Illiterate	10 (13.0)	30 (14.9)	31 (20.4)	71 (16.5)	
Primary, Intermediate school	52 (67.5)	140 (69.7)	102 (67.1)	294 (68.4)	
Secondary school and higher	15 (19.5)	31 (15.4)	19 (12.5)	65 (15.1)	
**Mother’s employment status**					0.969
No paid job/Housewife	75 (97.4)	192 (97.0)	147 (97.4)	414 (97.2)	
Paid job (daily/part-/full-time)	2 (2.6)	6 (3.0)	4 (2.6)	12 (2.8)	
**Father’s employment status**					0.627
No job/Part-time job	39 (52.7)	91 (46.4)	73 (49.7)	203 (48.7)	
Full-time job/ Self-employed	35 (47.3)	105 (53.6)	74 (50.3)	214 (51.3)	
**Monthly household income**					0.455
≤750,000 LBP	51 (71.8)	123 (63.7)	95 (64.6)	269 (65.5)	
>750,000 LBP	20 (28.2)	70 (36.3)	52 (35.4)	142 (34.5)	
**Length of stay in Lebanon (years)**	3.2 ± 1.9	4.0 ± 3.8	4.0 ±2.2	3.8 ±3.0	0.130
**UNHCR refugee registration status**					0.313
No	18 (23.7)	35 (17.4)	23 (15.5)	76 (17.9)	
Yes	58 (76.3)	166 (82.6)	125 (84.5)	349 (82.1)	
If yes, received WFP food assistance (e-vouchers) *					0.784
No	53 (91.4)	151 (91.5)	112 (90.3)	316 (91.1)	
Yes	5 (8.6)	14 (8.5)	12 (9.7)	31 (8.9)	
**Receipt of any cash or food assistance**					0.243
No assistance	68 (88.3)	180 (88.7)	125 (82.8)	373 (86.5)	
Any type of assistance	9 (11.7)	23 (11.3)	26 (17.2)	58 (13.5)	
**Reliance on savings or subsidies**					0.251
No reliance	76 (98.7)	192 (94.6)	147 (96.7)	415 (96.1)	
Any type of savings/subsidies	1 (1.3)	11 (5.4)	5 (3.3)	17 (3.9)	
**Perception of safety**					**0.012**
Very to reasonably safe	60 (77.9)	151 (75.1)	95 (62.5)	306 (71.2)	
Somewhat to very unsafe	17 (22.1)	50 (24.9)	57 (37.5)	124 (28.8)	
**Household characteristics**					
**Household type**					0.187
Nuclear family	37 (48.1)	102 (50.0)	89 (58.6)	228 (52.7)	
Extended family	40 (51.9)	102 (50.0)	63 (41.4)	205 (47.3)	
**Decision-making/Head of household**					0.199
Father/ Family-in-law	75 (97.4)	194 (97.5)	141 (94.0)	410 (96.2)	
Mother/ Both parents	2 (2.6)	2 (2.5)	9 (6.0)	16 (3.8)	
**Crowding index score**	4.0 ±2.0	3.6 ±1.6	3.7 ±1.5	3.7 ±1.6	0.189
**Number of children < 5 years per household**	2.4 ±1.8	2.7 ±1.5	2.8 ±1.5	2.7 ±1.5	0.327 **0.024**
1 to 2	53 (68.8)	104 (51.2)	75 (49.7)	232 (53.8)	
3 to 4	14 (18.2)	76 (37.4)	54 (35.8)	144 (33.4)	
≥5	10 (13.0)	23 (11.3)	22 (14.6)	55 (12.8)	

PM: Pregnant mothers; LM: Lactating mothers; NPNLM: Non-pregnant non-lactating mothers; ^a^ Categorical variables are expressed as *n*(%) and continuous variables are expressed as mean ± SD. ^b^ Lack of corresponding sum of frequencies with total sample size is due to missing data. ^c^ Significantly different at *p*-value < 0.05 (in bold); *p*-value was derived using one-way ANOVA test for continuous variables and chi-square analysis for categorical variables. * One mother reported receiving food assistance from another source and was not registered with the UNHCR.

**Table 2:** Healthcare service utilization and health characteristics by the reproductive status of Syrian refugee mothers.

Variables ^a^	PM *n* = 77	LM *n* = 204	NPNLM *n* = 152	Total ^b^ *n* = 433	*p*-Value ^c^
**Healthcare service utilization**					
**Health insurance**					0.493
Uninsured	70 (92.1)	190 (93.6)	143 (94.7)	403 (93.7)	
Public insurance	2 (2.6)	3 (1.5)	0 (0.0)	5 (1.2)	
Other	4 (5.3)	10 (4.9)	8 (5.3)	22 (5.1)	
**Type of healthcare usually sought**					0.287
Public healthcare only	51 (66.2)	147 (73.1)	99 (66.0)	297 (69.4)	
Public, private, and/or other healthcare	26 (33.8)	54 (26.9)	51 (34.0)	131 (30.6)	
**Number of antenatal care visits**					0.699
0 times	16 (21.1)	32 (16.1)	23 (15.1)	71 (16.6)	
1 to 3 times	17 (22.4)	40 (20.1)	34 (22.4)	91 (21.3)	
≥4 times	43 (56.6)	127 (63.8)	95 (62.5)	265 (62.1)	
**Sources of health and nutrition messages**					0.538
Healthcare professionals exclusively ^d^	13 (16.9)	30 (14.7)	17 (11.3)	60 (13.9)	
Family, friends, media, and/or other	51 (66.2)	126 (61.8)	96 (63.6)	273 (63.2)	
Multiple sources ^e^	13 (16.9)	48 (23.5)	38 (25.2)	99 (22.9)	
**Maternal health characteristics**					
**Self-reported previous diagnosis with anemia**					**0.040**
No	16 (21.1)	66 (32.8)	57 (37.7)	139 (32.5)	
Yes	60 (78.9)	135 (67.2)	94 (62.3)	289 (67.5)	
**Suffering from a flu (self-reported)**					**0.030**
No	66 (88.0)	178 (88.6)	145 (96.0)	389 (91.1)	
Yes	9 (12.0)	23 (11.4)	6 (4.0)	38 (8.9)	
**Suffering from symptoms ^f^**					0.438
No	10 (13.0)	29 (14.3)	16 (10.8)	55 (12.9)	
1 to 2 symptoms	24 (31.2)	77 (37.9)	47 (31.8)	148 (34.6)	
≥3 symptoms	43 (55.8)	97 (47.8)	85 (57.4)	225 (52.6)	
**Health problems during previous pregnancy ^g^**					0.537
No health problem	45 (58.4)	100 (49.5)	86 (57.0)	231 (53.7)	
1 health problem	26 (33.8)	82 (40.6)	50 (33.1)	158 (36.7)	
≥2 health problems	6 (7.8)	20 (9.9)	15 (9.9)	41 (9.5)	
**Mother’s use of micronutrient supplements**					
No	29 (37.7)	145 (72.9)	125 (82.2)	299 (69.9)	**<0.001**
Yes	48 (62.3)	54 (27.1)	27 (17.8)	129 (30.1)	
**If yes, what kind of supplement?**					
Iron/Iron-folic acid	33 (68.8)	32 (62.7)	18 (66.7)	83 (65.9)	0.816 0.210 0.068 **0.006** 0.234
Multivitamins	14 (29.2)	8 (15.7)	8 (29.6)	30 (23.8)	
Calcium	9 (18.8)	15 (29.4)	2 (7.4)	26 (20.6)	
Vitamin D	1 (2.1)	3 (5.9)	6 (22.2)	10 (7.9)	
Other	6 (12.5)	4 (7.8)	3 (11.1)	13 (10.3)	
**If yes, in what frequency?**					
Not compliant	3 (10.7)	11 (25.6)	3 (20.0)	17 (19.8)	0.307
Weekly or daily	25 (89.3)	32 (74.4)	12 (80.0)	69 (80.2)	

PM: Pregnant mothers; LM: Lactating mothers; NPNLM: Non-pregnant non-lactating mothers; ^a^ Categorical variables are expressed as *n*(%). ^b^ Lack of corresponding sum of frequencies with total sample size is due to missing data. ^c^ Significantly different at *p*-value < 0.05 (in bold); *p*-value was derived chi-square analysis for categorical variables. ^d^ Healthcare professionals included physicians, nurses, dietitians, and pharmacists. ^e^ Multiple sources include family, friends, media, other, and healthcare professionals. ^f^ Symptoms: headache (59.3%), fatigue (58.4%), dizziness (45.8%), loss of appetite (32.0%), insomnia (31.8%), shortness of breath (26.9%), difficulty concentrating (29.2%), pale skin (15.9%). ^g^ Health problems: anemia (23.5%), hypertension (10.5%), hemorrhage (5.6%), diabetes (3.0%), other problems (14.2%).

**Table 3:** Maternal lifestyle characteristics by the reproductive status of Syrian refugee mothers.

Variables ^a^	PM *n* = 77	LM *n* = 204	NPNLM *n* = 152	Total ^b^ *n* = 433	*p*-Value ^c^
**Maternal daily meal pattern**					
**Have breakfast every day**					**0.022**
No	20 (26.0)	51 (25.2)	57 (38.3)	128 (29.9)	
Yes	57 (74.0)	151 (74.8)	92 (61.7)	300 (70.1)	
**Main meals per day**	2.1 ± 0.6	2.2 ± 0.6	2.0 ± 0.6	2.1 ± 0.6	**0.018**
**Snacks per day**	1.5 ± 1.1	1.3 ± 1.0	1.2 ± 1.0	1.3 ± 1.0	0.155
**Drinks coffee/tea between meals ^d^**					0.065
No	55 (72.4)	118 (59.3)	84 (56.8)	257 (60.8)	
Yes	21 (27.6)	81 (40.7)	64 (43.2)	166 (39.2)	
**Drinks coffee/tea during a meal ^e^**					0.122
No	33 (43.4)	75 (37.7)	72 (48.6)	180 (42.6)	
Yes	43 (56.6)	124 (62.3)	76 (51.4)	243 (57.3)	
**Lifestyle characteristics**					
**Physical activity ^f^**					**0.033**
Low-intensity activity	27 (35.1)	85 (41.7)	40 (26.3)	152 (35.1)	
Moderate-intensity activity	21 (27.3)	56 (27.5)	44 (28.9)	121 (27.9)	
High-intensity activity	29 (37.7)	63 (30.9)	68 (44.7)	160 (37.0)	
**Currently smoking cigarettes or shisha**					**0.001**
No	68 (88.3)	171 (83.8)	105 (70.5)	344 (80.0)	
Yes	9 (11.7)	33 (16.2)	44 (29.5)	86 (20.0)	
**Currently drinking any type of alcohol**					0.260
No	77 (100.0)	194 (96.5)	144 (97.3)	415 (97.4)	
Yes	0 (0.0)	7 (3.5)	4 (2.7)	11 (2.6)	

PM: regnant mothers; LM: Lactating mothers; NPNLM: Non-pregnant non-lactating mothers; ^a^ Categorical variables are expressed as *n* (%) and continuous variables are expressed as mean ± SD. ^b^ Lack of corresponding sum of frequencies with total sample size is due to missing data. ^c^ Significantly different at *p*-value < 0.05 (in bold); *p*-value was derived chi-square analysis for categorical variables. ^d^ Between meals is defined as two hours before or after a meal. ^e^ During the course of a meal is defined as during or one hour before or after a meal. ^f^ Physical activity was measured using the IPAQ short form. Low-intensity was based on no reported activity or some activity not enough to meet the moderate- or high-intensity. Moderate-intensity was calculated as five or more days of any combination of walking, moderate- or vigorous-intensity activities achieving a minimum of at least 600 MET-minutes/week. High-intensity was calculated as seven or more days of any combination of walking, moderate- or vigorous-intensity activities accumulating at least 3000 MET-minutes/week [29].

**Table 4:** Anemia and nutritional status of Syrian refugee mothers and their children by the reproductive status.

Variables ^a^	PM *n* = 77	LM *n* = 204	NPNLM *n* = 152	Total ^b^ *n* = 433	*p*-Value ^c^
**Anemia among mothers**					
**Hemoglobin concentration (g/dL)**	11.8 ± 1.3	12.9 ± 1.1	12.7 ± 1.3	12.6 ± 1.3	**<0.001**
**Total anemia ^d^**					0.199
Not anemic	63 (81.8)	162 (80.6)	111 (73.5)	336 (78.3)	
Anemic	14 (18.2)	39 (19.4)	40 (26.5)	93 (21.7)	
**Classification of anemia ^e^**					**0.043**
Not anemic	63 (81.8)	162 (80.6)	111 (73.5)	336 (78.3)	
Mild anemia	6 (7.8)	32 (15.9)	28 (18.5)	66 (15.4)	
Moderate anemia	8 (10.4)	7 (3.5)	12 (7.9)	27 (6.3)	
**Anemia among children**					
**Hemoglobin concentration (g/dL)**	11.7 ± 1.3	11.3 ± 1.2	11.6 ± 1.2	11.4 ± 1.3	**0.008**
**Total anemia (0–59 months) ^d^**					0.060
Not anemic	60 (77.9)	130 (64.4)	109 (72.2)	299 (69.5)	
Anemic	17 (22.1)	72 (35.6)	42 (27.8)	131 (30.5)	
**Classification of anemia (6–59 months; *n* = 318) ^e^**					**0.001**
Not anemic	57 (78.1)	52 (50.0)	101 (71.6)	210 (66.0)	
Mild anemia	10 (13.7)	39 (37.5)	28 (19.9)	77 (24.2)	
Moderate anemia	6 (8.2)	13 (12.5)	12 (8.5)	31 (9.8)	
**Nutritional status of mothers**					
**MUAC (cm)**	28.6 ± 3.9	29.4 ± 4.1	29.9 ± 4.8	29.4 ± 4.4	0.148
**Classification of malnutrition using MUAC**					0.056
Undernourished (<23.0 cm)	2 (2.6)	8 (3.9)	10 (6.6)	20 (4.6)	
Normal weight (23.0–27.9 cm)	32 (42.1)	72 (35.5)	42 (27.6)	146 (33.9)	
Overweight (28.0–30.9 cm)	25 (32.9)	54 (26.6)	37 (24.3)	116 (26.9)	
Obese (≥31.0 cm)	17 (22.4)	69 (34.0)	63 (41.4)	149 (34.6)	
**Nutritional status of non-pregnant mothers ^f^**					
**BMI**					0.142
Underweight (<18.5 kg/m^2^)	-	2 (1.0)	7 (4.6)	9 (2.5)	
Normal weight (18.5–24.9 kg/m^2^)		76 (37.3)	55 (36.2)	131 (36.8)	
Overweight (25.0–29.9 kg/m^2^)		68 (33.3)	43 (28.3)	111 (31.2)	
Obese (≥30.0 kg/m^2^)		58 (28.4)	47 (30.9)	105 (29.5)	
**Waist circumference**					0.362
Normal (≤79 cm)	-	67 (32.8)	57 (37.5)	124 (34.8)	
At-risk (>80 cm)		137 (67.2)	95 (62.5)	232 (65.2)	
**Waist**–**hip ratio**					0.066
Normal (<0.85 cm)	-	94 (46.1)	85 (55.9)	179 (50.3)	
Substantially increased risk (≥0.85 cm)		110 (53.9)	67 (44.1)	177 (49.7)	
**Nutritional status of the children (0** **–59 months)**					
**Length/Height-for-age Z-score (L/HAZ)**					0.458
No stunting (L/HAZ ≥ −2)	69 (90.8)	189 (92.6)	135 (88.8)	393 (91.0)	
Stunting (L/HAZ < −2)	7 (9.2)	15 (7.4)	17 (11.2)	39 (9.0)	
**Weight-for-age Z-score (WAZ)**					0.327
No underweight (WAZ ≥ −2)	73 (96.1)	192 (94.1)	148 (97.4)	413 (95.6)	
Underweight (WAZ < −2)	3 (3.9)	12 (5.9)	4 (2.6)	19 (4.4)	
**Weight-for-length/height Z-score (WHZ)**					0.050
No wasting (WHZ ≥ −2)	69 (90.8)	191 (94.1)	149 (98.0)	409 (94.9)	
Wasting (WHZ < −2)	7 (9.2)	12 (5.9)	3 (2.0)	22 (5.1)	
**BMI-for-age Z-score (BAZ)**					0.086
Wasting (BAZ < −2)	7 (9.2)	13 (6.4)	2 (1.3)	22 (5.1)	
Normal weight (−2 ≤ BAZ ≤ +2)	66 (86.8)	181 (88.7)	144 (94.7)	391 (90.5)	
Overweight/obese (BAZ > +2)	3 (3.9)	10 (4.9)	6 (3.9)	19 (4.4)	

PM: Pregnant mothers; LM: Lactating mothers; NPNLM: Non-pregnant non-lactating mothers; ^a^ Categorical variables are expressed as *n* (%) and continuous variables are expressed as mean ± SD. ^b^ Lack of corresponding sum of frequencies with total sample size is due to missing data. ^c^ Significantly different at *p*-value < 0.05 (in bold); *p*-value was derived using one-way ANOVA test for continuous variables and chi-square analysis for categorical variables. ^d^ Total anemia was defined as hemoglobin (Hb) < 12.0 g/dL for LM and NPNLM, <11.0 g/dL for PM and children (6–59 months), and <10.5 g/dL for infants (0–5 months). ^e^ Anemia was classified as mild and moderate for LM and NPNLM (Hb 11.0–11.9 g/dL and 8.0–10.9 g/dL, respectively) and for PM and children aged 6 to 59 months (Hb 10.0–10.9 g/dL and 7.0–9.9 g/dL, respectively). ^f^ Non-pregnant mothers include lactating mothers and non-pregnant non-lactating mothers.

**Table 5:** Odds ratio of anemia, nutritional status, and age by the reproductive status of mothers.

Variables	OR (95% CI) ^a^ Reference Group: NPNLM		aOR (95% CI) ^b^ Reference Group: NPNLM	
	Pregnant mothers	Lactating mothers	Pregnant mothers	Lactating mothers
**Age**				
**Age of the mother (years)**	0.95 (0.91–0.99) *	0.94 (0.91–0.98) **	0.92 (0.86–0.99) *	0.97 (0.91–1.05)
**Age of the children (months)**	0.99 (0.97–1.00)	0.83 (0.79–0.86) ^α^	1.01 (0.98–1.03)	0.82 (0.78–0.86) ^α^
**Health and lifestyle characteristics**				
**Self-reported previous diagnosis with anemia**				
No	1.0	1.0	1.0	1.0
Yes	2.27 (1.20–4.32) *	1.24 (0.80–1.93)	2.60 (1.21–5.55) *	1.75 (0.90–3.40)
**Suffering from the flu (self-reported)**				
No	1.0	1.0	1.0	1.0
Yes	3.30 (1.13–9.64) *	3.12 (1.24–7.87) *	3.66 (1.08–12.42) *	2.63 (0.72–9.58)
**Mother’s use of supplements**				
No	1.0	1.0	1.0	1.0
Yes	7.66 (4.12–14.26) ^α^	1.72 (1.02–2.90) *	12.47 (5.64–27.55) ^α^	2.37 (1.05–5.35) *
**Have breakfast every day**				
No	1.0	1.0	1.0	1.0
Yes	1.77 (0.96–3.24)	1.83 (1.16–2.90) **	2.14 (1.05–4.40) *	2.28 (1.14–4.56) *
**Currently smokes cigarettes or shisha**				
No	1.0	1.0	1.0	1.0
Yes	0.32 (0.14–0.69) **	0.46 (0.28–0.77) **	0.34 (0.14–0.83) *	0.41 (0.20–0.86) *
**Anemia and nutritional status of mothers**				
**Classification of anemia ^c^**				
Not anemic	1.0	1.0	1.0	1.0
Mild anemia	0.38 (0.15–0.96) *	0.78 (0.45–1.37)	0.26 (0.08–0.82) *	1.09 (0.43–2.71)
Moderate anemia	1.18 (0.46–3.03)	0.40 (0.15–1.05)	2.75 (0.70–10.84)	0.93 (0.20–4.45)
**Classification of malnutrition using MUAC**				
Normal weight (23.0–27.9 cm)	1.0	1.0	1.0	1.0
Undernourished (<23.0 cm)	0.26 (0.05–1.28)	0.47 (0.17–1.27)	0.26 (0.05–1.50)	0.36 (0.09–1.48)
Overweight (28.0–30.9 cm)	0.89 (0.45–1.76)	0.85 (0.48–1.50)	0.82 (0.36–1.86)	0.94 (0.41–2.16)
Obese (≥31.0 cm)	0.35 (0.18–0.72) **	0.64 (0.38–1.06)	0.25 (0.10–0.60)**	0.86 (0.39–1.92)
**Anemia and nutritional status of children**				
Total anemia (0–59 months) ^c^				
Not anemic	1.0	1.0	1.0	1.0
Anemic	0.74 (0.39–1.40)	1.44 (0.91–2.27)	0.66 (0.30–1.45)	2.11 (1.07–4.17) *
**Classification of anemia (6–59 months) ^d^**				
Not anemic	1.0	1.0	1.0	1.0
Mild anemia	0.63 (0.29–2.49)	2.70 (1.50–4.88) **	0.75 (0.21–2.62)	2.98 (1.26–7.04) **
Moderate anemia	0.89 (0.32–1.40)	2.10 (0.90–4.94)	0.52 (0.20–1.36)	2.67 (0.83–8.57)
**BMI-for-age Z-score (BAZ) (0–59 months)**				
Normal weight (−2 ≤ BAZ ≤ +2)	1.0	1.0	1.0	1.0
Wasting (BAZ < −2)	7.64 (1.54–37.76) *	5.17 (1.15–23.28) *	5.90 (1.02–34.18) *	2.37 (0.44–11.90)
Overweight/obese (BAZ > +2)	1.09 (0.26–4.50)	1.33 (0.47–3.73)	3.01 (0.54–16.79)	0.58 (0.07–4.82)

NPNLM: Non-pregnant non-lactating mothers; * *p* < 0.05, ** *p* < 0.01, ^α^ *p* < 0.001; ^a^ Odds Ratio (OR) of the dependent variable (NPNLM vs. Pregnant mothers and Lactating mothers) are presented with 95% CI using simple logistic regression. ^b^ Adjusted OR (aOR) are presented with 95% CI using multiple logistic regression analysis. Model 1: adjusted for age of the mother and child, crowding index, and the total number of under-five children per household as continuous variables and for the sex of the child, education levels of the mother and father, employment status of the father, household monthly income, UNHCR registration status, head of household, household type, perception of safety, receipt of cash or food assistance, reliance on savings, and healthcare type as categorical variables. ^c^ Total anemia was defined as hemoglobin (Hb) < 12.0 g/dL for LM and NPNLM, <11.0 g/dL for PM and children (6–59 months), and <10.5 g/dL for infants (0–5 months). ^d^ Anemia was classified as mild and moderate for LM and NPNLM (Hb 11.0–11.9 g/dL and 8.0–10.9 g/dL, respectively) and for PM and children aged 6 to 59 months (Hb 10.0–10.9 g/dL and 7.0–9.9 g/dL, respectively).

**Table 6:** Key characteristics and nutritional status indicators according to total anemia among Syrian refugee mothers.

Variables ^a^	Not Anemic	Anemic	Maternal Anemia	
**Socio-economic and household characteristics (*n* = 433)**	***n*** **= 334**	***n*** **= 93**	**OR (95% CI) ^b^**	**aOR (95% CI) ^c^**
**Crowding index score**	3.6 ±1.5	4.1 ±2.0	1.2 (1.03–1.34) *	1.09 (0.88–1.35)
**Receipt of WFP food assistance (e-vouchers)**				
No	311 (94.0)	77 (87.5)	1.0	1.0
Yes	20 (6.0)	11 (12.5)	2.22 (1.02–4.83) *	1.56 (0.55–4.44) ^d^
**Number of children < 5 years per household**				
1 to 2	188 (56.0)	41 (45.1)	1.0	1.0
3 to 4	115 (34.2)	28 (30.8)	1.12 (0.66–1.90)	0.62 (0.28–1.39)
≥5	33 (9.8)	22 (24.2)	3.06 (1.62–5.78)**	1.73 (0.58–5.10)
**Health characteristics of mothers (*n* = 433)**	***n*** **= 334**	***n*** **= 93**	**OR (95% CI) ^b^**	**aOR (95% CI) ^c^**
**Self-reported previous diagnosis with anemia**				
No	118 (35.6)	20 (21.5)	1.0	1.0
Yes	213 (64.4)	73 (78.5)	2.02 (1.17–3.48) *	2.27 (1.14–4.52) *
**Sources of health and nutrition messages**				
Healthcare professionals exclusively ^e^	38 (11.3)	22 (23.9)	1.0	1.0
Family, friends, media, and/or other	219 (65.2)	52 (56.5)	0.41 (0.22–0.75) **	0.42 (0.19–0.94) *
Multiple sources ^f^	79 (23.5)	18 (19.6)	0.39 (0.19–0.82) *	0.23 (0.08–0.66) **
**Anemia status among children (0–59 months) (*n* = 433)**	***n*** **= 333**	***n*** **= 93**	**OR (95% CI) ^b^**	**aOR (95% CI) ^c^**
**Total anemia (0–59 months) ^g^**				
Not anemic	239 (71.8)	56 (60.2)	1.0	1.0
Anemic	94 (28.2)	37 (39.8)	1.68 (1.04–2.71) *	2.67 (1.42–5.02) **
**Classification of anemia (6–59 months) (*n* = 318)**	***n*** **= 242**	***n*** **= 75**	**OR (95% CI) ^b^**	**aOR (95% CI) ^c^**
**Classification of anemia (6–59 months) ^h^**				
Not anemic	165 (68.2)	44 (58.7)	1.0	1.0
Mild anemia	55 (22.7)	22 (29.3)	1.50 (0.83–2.72)	4.40 (1.79–10.80) ^α^
Moderate anemia	22 (9.1)	9 (12.0)	1.53 (0.66–3.57)	2.39 (0.72–7.97)
**Nutritional status of non-pregnant mothers ^i^ (*n* = 356)**	***n*** **= 273**	***n*** **= 79**	**OR (95% CI) ^b^**	**aOR (95% CI) ^j^**
**Waist circumference**				
Normal	104 (38.1)	20 (25.3)	1.0	1.0
At-risk (>80 cm)	169 (61.9)	59 (74.7)	1.82 (1.03–3.19) *	3.05 (1.34–6.92) **
**Nutritional status of NPNLM (*n* = 152)**	***n*** **= 111**	***n*** **= 40**	**OR (95% CI) ^b^**	**aOR (95% CI) ^k^**
**Waist circumference**				
Normal	49 (44.1)	8 (20.0)	1.0	1.0
At-risk (>80 cm)	62 (55.9)	32 (80.0)	3.16 (1.34–7.48) **	10.90 (2.74–43.38)**
**BMI for the mother**				
Normal weight	46 (41.4)	9 (22.5)	1.0	1.0
Underweight	4 (3.6)	3 (7.5)	3.83 (0.73–20.13)	13.39 (1.52–118.12) *
Overweight/Obese	61 (55.0)	28 (70.0)	2.35 (1.01–5.45) *	14.68 (3.22–66.96) **
**Consumption of coffee and tea (*n* = 375)**	***n*** **= 294**	***n*** **= 81**	**OR (95% CI) ^b^**	**aOR (95% CI) ^c^**
**Drinks coffee/tea between meals ^l^**				
No	140 (47.6)	35 (43.2)	1.0	1.0
Yes	154 (52.4)	46 (56.8)	0.82 (0.48–1.39)	0.87 (0.44–1.73)
**Drinks coffee/tea during a meal ^m^**				
No	140 (47.6)	35 (43.2)	1.0	1.0
Yes	154 (52.4)	46 (56.8)	1.20 (0.73–1.96)	1.38 (0.73–2.62)

NPNLM: Non-pregnant non-lactating mothers; * *p* < 0.05, ** *p* < 0.01, ^α^ *p* < 0.001; ^a^ Categorical variables are expressed as *n*(%) and continuous variables are expressed as mean ± SD. Lack of corresponding sum of frequencies with total sample size is due to missing data. ^b^ Odds Ratio (OR) of the dependent variable (Anemic vs. Not Anemic (reference group)) are presented with 95% CI using simple logistic regression. ^c^ Adjusted OR (aOR) are presented with 95% CI using multiple logistic regression analysis. Model 2: adjusted for age of the mother and child, sex of the child, reproductive status of the mother (pregnant, lactating, or non-pregnant non-lactating), marital status, education and employment status of the parents, household monthly income, crowding index, UNHCR registration status, perception of safety, receipt of cash or food assistance, reliance on savings, household type, head of household, total number of under-five children, health insurance coverage, healthcare type, and sources of health messages. ^d^ Model 3: Model 2 without receipt of cash or food assistance. ^e^ Healthcare professionals included physicians, nurses, dietitians, and pharmacists. ^f^ Multiple sources include family, friends, media, other, and healthcare professionals. ^g^ Total anemia was defined as hemoglobin (Hb) < 12.0 g/dL for LM and NPNLM, <11.0 g/dL for PM and children (6–59 months), and <10.5 g/dL for infants (0–5 months). ^h^ Anemia was classified as mild and moderate for LM and NPNLM (Hb 11.0–11.9 g/dL and 8.0–10.9 g/dL, respectively) and for PM and children aged 6 to 59 months (Hb 10.0–10.9 g/dL and 7.0–9.9 g/dL, respectively). ^i^ Non-pregnant mothers include lactating mothers and non-pregnant non-lactating mothers. ^j^ Model 4: Model 2 without reproductive status of the mother (pregnant, lactating, or non-pregnant non-lactating). ^k^ Model 5: Model 2 without the marital status, employment status of the father, UNHCR registration status, reliance on savings, and reproductive status of the mother (pregnant, lactating, or non-pregnant non-lactating). ^l^ Between meals is defined as two hours before or after a meal. ^m^ During the course of a meal is defined as during or one hour before or after a meal.

**Table 7:** Dietary intake and nutritional inadequacy (<2/3rd of DRIs) of energy and macro- and micronutrients by the reproductive status of Syrian mothers.

Variables	Intake/Day (Mean ± SD)				<2/3rd DRIs (*n*, %)			
Energy and Macronutrients (%EI)	PM *n* = 77	LM *n* = 204	NPNLM *n* = 152	Total *n* = 433	PM *n* = 77	LM *n* = 204	NPNLM *n* = 152	Total *n* = 433
Energy (kcal/d)	1622.6 ± 872.9 ^a^	1555.5 ± 729.4 ^b^	1341.5 ± 685.2 ^c^	1492.3 ± 749.0	-	-	-	-
Carbohydrates (%EI/d)	51.8 ± 10.6	51.9 ± 11.2	53.1 ± 11.7	52.3 ± 11.3	-	-	-	-
Protein (%EI/d)	12.2 ± 5.1	12.3 ± 3.9	12.8 ± 4.6	12.4 ± 4.4	-	-	-	-
Total fat (%EI/d)	37.2 ± 11.0	36.8 ± 11.3	35.5 ± 11.3	36.4 ± 11.2	-	-	-	-
MUFA (%EI/d)	13.5 ± 8.4	12.8 ± 8.0	12.6 ± 9.4	12.9 ± 8.6	-	-	-	-
Linoleic acid (%EI/d)	8.9 ± 4.3	9.0 ± 5.1	8.7 ± 4.7	8.9 ± 4.8	-	-	-	-
Linolenic acid (%EI/d)	1.6 ± 3.8	1.4 ± 2.7	1.4 ± 2.8	1.4 ± 3.0	-	-	-	-
PUFA (%EI/d)	12.2 ± 13.0	12.1 ± 14.3	12.7 ± 21.3	12.3 ± 16.9	-	-	-	-
SFA (%EI/d)	9.4 ± 10.7	9.2 ± 7.0	8.2 ± 4.2	8.9 ± 7.0	-	-	-	-
TFA (%EI/d)	0.1 ± 0.22	0.2 ± 0.4	0.2 ± 0.4	0.2 ± 0.3	-	-	-	-
Cholesterol (mg/d)	146.4 ± 158.0 ^a^	162.5 ± 186.5 ^b^	116.9 ± 130.7 ^c^	143.6 ± 164.7	-	-	-	-
Total sugar (%EI/d)	12.8 ± 8.5	13.8 ± 7.79	15.0 ± 7.8	14.0 ± 8.0	-	-	-	-
**Macronutrient intake**								
Carbohydrates (g/d)	205.9 ± 104.8 ^a^	197.0 ± 90.6 ^b^	171.4 ± 81.4 ^c^	189.6 ± 91.1	16 (20.8) ^d^	60 (29.4) ^e^	21 (13.8) ^f^	97 (22.4)
Protein (g/d)	45.3 ± 21.7	46.7 ± 26.3	41.4 ± 24.5	44.6 ± 25.0	46 (59.7) ^d^	121 (59.3) ^e^	58 (38.2) ^f^	225 (52.0)
Linoleic acid (g/d)	17.1 ± 12.5	16.2 ± 12.7	13.9 ± 11.2	15.6 ± 12.2	24 (31.2)	65 (31.9)	55 (36.4)	144 (33.3)
Linolenic acid (g/d)	4.3 ± 13.2	2.3 ± 4.4	2.2 ± 4.8	2.6 ± 6.9	43 (55.8)	110 (53.9)	90 (59.2)	243 (56.1)
Fibers (g/d)	16.8 ± 12.0	15.5 ± 11.0	14.5 ± 10.7	15.3 ± 11.1	51 (66.2) ^d^	148 (72.5) ^e^	107 (70.9) ^f^	306 (70.8)
**Micronutrient intake**								
Iron (mg/d)	10.2 ± 9.8	8.2 ± 6.2	8.0 ± 6.1	8.5 ± 7.0	63 (81.8) ^d^	92 (45.1) ^e^	121 (80.1) ^f^	276 (63.9)
Folate (µg/d)	238.8 ± 222.0	221.3 ± 204.5	203.8 ± 178.3	218.2 ± 199.0	65 (84.4)	161 (78.9)	116 (76.3)	342 (79.0)
Vitamin B12 (µg/d)	1.4 ± 3.3	1.4 ± 2.4	1.2 ± 1.8	1.4 ± 2.4	59 (76.6)	160 (78.4)	117 (77.0)	336 (77.6)
Vitamin C (mg/d)	62.9 ± 65.1	64.4 ± 58.6	52.9 ± 43.0	60.1 ± 55.1	46 (59.7)	140 (68.6)	86 (57.0)	272 (63.0)
Vitamin A (µg/d)	418.6 ± 668.8	425.6 ± 530.7	329.8 ± 397.3	390.7 ± 517.9	57 (74.0) ^d^	182 (89.2) ^e^	126 (82.9) ^f^	365 (84.3)
Vitamin D (µg/d)	0.7 ± 0.8 ^a^	0.8 ± 1.1 ^b^	0.4 ± 0.6 ^c^	0.6 ± 0.9	77 (100.0)	204 (100.0)	152 (100.0)	433 (100.0)
Vitamin E (mg/d)	9.1 ± 9.2	7.7 ± 5.9	7.1 ± 6.5	7.8 ± 6.8	53 (68.8) ^d^	175 (85.8) ^e^	120 (78.9) ^f^	348 (80.4)
Vitamin K (µg/d)	132.1 ± 194.5	133.6 ± 276.9	111.0 ± 174.7	125.4 ± 231.2	35 (45.5)	90 (44.1)	84 (55.6)	209 (48.4)
Thiamin (mg/d)	1.3 ± 0.8 ^a^	1.3 ± 0.7 ^b^	1.1 ± 0.6 ^c^	1.2 ± 0.7	24 (31.2)	65 (31.9)	40 (26.5)	129 (29.9)
Riboflavin (mg/d)	1.1 ± 0.7 ^a^	1.1 ± 0.6 ^b^	0.9 ± 0.5 ^c^	1.0 ± 0.6	37 (48.1)	111 (54.4)	70 (46.4)	218 (50.5)
Niacin (mg/d)	13.7 ± 7.1	13.8 ± 8.3	11.9 ± 6.9	13.1 ± 7.6	35 (45.5)	86 (42.2)	63 (41.4)	184 (42.5)
Pantothenic acid (mg/d)	3.4 ± 2.4	3.3 ± 2.1	2.9 ± 1.9	3.2 ± 2.1	55 (71.4) ^d^	171 (83.8) ^e^	111 (73.0) ^f^	337 (77.8)
Vitamin B6 (mg/d)	1.0 ± 0.6 ^a^	0.9 ± 0.6 ^b^	0.8 ± 0.5 ^c^	0.9 ± 0.6	52 (67.5) ^d^	173 (84.8) ^e^	99 (65.6) ^f^	324 (75.0)
Zinc (mg/d)	6.4 ± 4.3	6.0 ± 3.7	5.5 ± 3.5	5.9 ± 3.8	55 (71.4) ^d^	161 (78.9) ^e^	94 (62.3) ^f^	310 (71.8)
Copper (mg/d)	1.3 ± 1.1 ^a^	1.0 ± 0.6 ^b^	0.9 ± 0.6 ^c^	1.0 ± 0.7	20 (26.0) ^d^	98 (48.0) ^e^	58 (38.2) ^f^	176 (40.6)
Calcium (mg/d)	475.1 ± 386.1	449.6 ± 341.2	414.1 ± 325.9	441.7 ± 344.3	62 (80.5)	166 (81.4)	125 (82.8)	353 (81.7)
Magnesium (mg/d)	216.2 ± 140.9 ^a^	183.1 ± 99.6 ^b^	174.2 ± 105.4 ^c^	186.8 ± 110.7	52 (67.5)	139 (68.1)	108 (71.5)	299 (69.2)
Sodium (mg/d)	2088.9 ± 1235.6	2217.8 ± 1335.6	1948.2 ± 1170.1	2100.2 ± 1265.0	13 (16.9)	33 (16.2)	32 (21.1)	78 (18.0)
Potassium (mg/d)	744.6 ± 459.1 ^a^	1798.2 ± 956.4 ^b^	1600.4 ± 879.9 ^c^	1752.9 ± 972.2	66 (85.7) ^d^	195 (95.6) ^e^	144 (94.7) ^f^	405 (93.5)
Phosphorus (mg/d)	744.6 ± 459.1	696.4 ± 437.9	621.4 ± 391.6	678.6 ± 427.6	20 (26.0)	66 (32.4)	61 (40.4)	147 (34.0)
Manganese (mg/d)	2.8 ± 1.6	2.5 ± 1.6	2.4 ± 1.5	2.5 ± 1.6	14 (18.2) ^d^	71 (34.8) ^e^	33 (21.9) ^f^	118 (27.3)
Selenium (µg/d)	69.9 ± 39.0 ^a^	72.0 ± 37.9 ^b^	59.2 ± 34.0 ^c^	67.2 ± 37.2	18 (23.4)	55 (27.0)	40 (26.3)	113 (26.1)

DRIs: Dietary Reference Intakes; PM: Pregnant mothers; LM: Lactating mothers; NPNLM: Non-pregnant non-lactating mothers; d: day; %EI: percent energy intake; MUFA: monounsaturated fatty acids; PUFA: polyunsaturated fatty acids; SFA: saturated fatty acids; TFA: trans-fatty acids; Categorical variables are expressed as *n* (%) and continuous variables are expressed as mean ±SD. The nutritional inadequacy (<2/3rd DRIs) represents the proportion of mothers not meeting 2/3rd of the RDA or AI for micronutrients key macronutrient according to their age group and reproductive status (displayed in Annex A—Table A1). ^a,b,c^ Mean values in a row with unlike superscript letters were significantly different (*p* < 0.05) using ANOVA test for the comparison of mean intakes/day. ^d,e,f^ Mean values in a row with unlike superscript letters were significantly different (*p* < 0.05) using chi-square analysis for <2/3rd DRI.

## Data Availability

Dataset analyzed during this study are available from the corresponding author on reasonable request.

## Appendix A

**Table A1:** Dietary Reference Intakes (DRIs) for macro- and micronutrients women of reproductive age according to their reproductive status.

	Pregnant Mothers	Lactating Mothers	Non-Pregnant Non-Lactating Mothers
**Energy and macronutrients**			
Energy (kcal/d)	2200–2652	2600–2700	2200
Carbohydrates (%EI/d)	45–65	45–65	45–65
Protein (%EI/d)	10–35	10–35	10–35
Total fat (%EI/d)	20–35	20–35	20–35
MUFA (%EI/d)	15–20	15–20	15–20
Linoleic acid (%EI/d)	5–10	5–10	5–10
Linolenic acid (%EI/d)	0.6–1.2	0.6–1.2	0.6–1.2
PUFA (%EI/d)	6–11	6–11	6–11
SFA (%EI/d)	8–10	8–10	8–10
TFA (%EI/d)	<1	<1	<1
Cholesterol (mg/d)	<200	<200	<200
Total sugar (%EI/d)	<10	<10	<10
**Macronutrients**			
Carbohydrates (g/d)	**175**	**210**	**130**
Protein (g/d)	**71**	**71**	**46**
Linoleic acid (g/d)	13 *	13 *	11–12 *
Linolenic acid (g/d)	1.4 *	1.3 *	1.1 *
Fibers (g/d)	28 *	29 *	25–26 *
**Micronutrients**			
Iron (mg/d)	**27**	**9–10**	**15–18**
Folate (µg/d)	**600**	**500**	**400**
Vitamin B12 (µg/d)	**2.6**	**2.8**	**2.4**
Vitamin C (mg/d)	**80–85**	**115–120**	**65–75**
Vitamin A (µg/d)	**750–770**	**1200–1300**	**700**
Vitamin D (µg/d)	**15**	**15**	**15**
Vitamin E (mg/d)	**15**	**19**	**15**
Vitamin K (µg/d)	75–90 *	75–90 *	75–90 *
Thiamin (mg/d)	**1.4**	**1.4**	**1–1.1**
Riboflavin (mg/d)	**1.4**	**1.6**	**1–1.1**
Niacin (mg/d)	**18**	**17**	**14**
Pantothenic acid (mg/d)	6 *	7 *	5 *
Vitamin B6 (mg/d)	**1.9**	**2**	**1.2–1.3**
Zinc (mg/d)	**11–12**	**12–13**	**8–9**
Copper (mg/d)	**1**	**1.3**	**0.9**
Calcium (mg/d)	**1000–1300**	**1000–1300**	**1000–1300**
Magnesium (mg/d)	**350–400**	**310–360**	**310–360**
Sodium (mg/d)	1500 *	1500 *	1500 *
Potassium (mg/d)	4700 *	5100 *	4700 *
Phosphorus (mg/d)	**700–1250**	**700–1250**	**700–1250**
Manganese (mg/d)	2.0 *	2.6 *	1.6–1.8 *
Selenium (µg/d)	**60**	**55**	**70**

d: day; %EI: percent energy intake; MUFA: monounsaturated fatty acids; PUFA: polyunsaturated fatty acids; SFA: saturated fatty acids; TFA: trans-fatty acids. Dietary Reference Intakes (DRIs) refers to the Recommended Dietary Allowances (RDAs), Adequate Intakes (AIs), and Acceptable Macronutrient Distribution Ranges (AMDRs) for energy and macro- and micronutrients as recommended by the Institute of Medicine [47,48]. RDAs are presented in **bold type** and AIs in ordinary types followed by an asterisk (*). Intakes of MUFA, PUFA, SFA, and TFA were analyzed based on the FAO recommendations [49]. Cholesterol intake was assessed based on the criteria of the National Institute of Health [50]. The reference intake of total sugar was based on the WHO recommendation [51].
